# A lack of evidence for disability-inclusive maternal health interventions and promising progress: an updated systematic review

**DOI:** 10.3389/fgwh.2025.1711871

**Published:** 2025-12-16

**Authors:** Alka Dev, Sophia E. Allen, Sophia Sisson

**Affiliations:** 1 The Dartmouth Institute for Health Policy and Clinical Practice, Geisel School of Medicine, Lebanon, NH, United States; 2 Department of Obstetrics and Gynecology, Dartmouth Health, Lebanon, NH, United States; 3 Georgia Health Policy Center, Georgia State University, Atlanta, GA, United States

**Keywords:** disability, pregnancy, maternal health, accommodations, co-production

## Abstract

**Background:**

Pregnant women with disabilities experience significantly higher rates of adverse pregnancy outcomes compared to those without disabilities. Evidence-based interventions that address disability-related barriers during pregnancy are essential to reducing health disparities.

**Objective:**

We aimed to update a 2014 systematic review to identify interventions designed for pregnant women with disabilities.

**Methods:**

We conducted a systematic review of studies published between 2012 and 2025 to identify interventions addressing disability-related barriers during pregnancy and birth.

**Results:**

We found a striking absence of evidence with no eligible studies identified from 22,719 publications. While we found multiple studies that evaluated the safety and efficacy of medications to manage disability-associated conditions during pregnancy, none of these studies focused on the potential disabling impact of the health conditions for pregnant women with disabilities, our intended focus. However, in our discussion, we describe three recent pilots, including co-produced resources for pregnant patients with disabilities, educational interventions for midwives, and an innovative patient empowerment tool, that suggest the field may be at a turning point.

**Conclusions:**

Our systematic review did not find evidence of disability inclusive maternal health interventions to improve pregnancy and childbirth experiences. However, we point to limited but promising studies for their use of co-production and patient engagement principles underscoring the potential for accelerating progress when research is conducted with, rather than on, disabled communities. While the pilots serve as proof of concept that disability-inclusive reproductive health research is both necessary and achievable, investments in disability inclusive maternal healthcare could yield significant returns for those with disabilities.

## Introduction

Disability encompasses physical, sensory, intellectual, and developmental conditions, along with a host of associated challenges in day-to-day functioning, health status, employment, and long-term survival, that make it difficult for individuals with disabilities to participate in certain activities and engage in their communities (1). During pregnancy, having a disability can make coordinating maternal healthcare especially challenging (2). Physical disability, for example, can complicate activities ranging from daily walking during pregnancy to giving birth. There is over a decade of evidence documenting adverse pregnancy and birth outcomes among women with disabilities compared to those without disabilities, including higher rates of pregnancy complications, severe maternal morbidity, preterm birth, low birthweight, and cesarean delivery (3–12). These disparities persist regardless of whether disability is identified using clinical evaluation or self-reported (8, 13). The disability community has consistently articulated barriers, including physical inaccessibility, financial need, provider bias, poor communication, and feelings of dehumanization, in their ability to access and benefit from maternal healthcare during pregnancy (8, 14–18).

The evidence of need is global. Women with disabilities have reported facing multiple barriers to accessing maternal healthcare from social stigma and judgement associated with pregnancy and being disabled to encountering clinical staff who lack knowledge or understanding of disability and appropriate communication skills across Austria (19), Brazil (20), Cambodia (21), Canada (22), Eswatini (23), Ghana (24), India (25), Nepal (26), Philippines (27), South Africa (28), the U.K. (29, 30), and the U.S (31, 32). Despite the potential adversities, statistical parity exists between women with and without disabilities in terms of pregnancy incidence, with empirical investigations both qualitative and quantitative, illustrating the potential for affirmative pregnancy experiences among individuals with disabilities (33, 34). However, there is limited evidence in the current literature on interventions improving pregnancy experiences and outcomes, especially supporting the whole person rather than only treating the disabling illness. A previous systematic review identified three interventions tailored to address disability within the context of pregnancy and the postpartum period, two of which focused on HIV positive mothers and their infants (35). In light of the recent American College of Obstetrics and Gynecology guidelines on accessible obstetric and gynecologic care for patients with disabilities (36), and the launch of the Africa region report on persistent gaps in inclusion, participation, and the protection of the rights of women with disabilities (37), we aimed to provide an updated synthesis of the literature interventions targeting pregnancy in individuals with disabilities.

## Methods

### Eligibility

This review included intervention studies with three target populations: (1) pregnant women with disabilities, (2) families, partners, and children of pregnant women with disabilities, and (3) healthcare professionals providing care to pregnant women with disabilities. We included randomized controlled trials, clustered randomized controlled trials, controlled clinical trials, quasi-experimental studies, and observational studies, including retrospective and prospective cohort studies. Eligible interventions aimed to improve pregnancy or birth outcomes by addressing disability-related barriers during the perinatal period. In keeping with the Malouf et al. review, we included search criteria provided by the authors, and added conditions from more recent maternal disability literature (Supplementary Appendix A1) (4). To be eligible, the study must have aimed to implement an intervention focused on pregnant women with disabilities or their families. The intervention must have occurred during pregnancy or childbirth.

However, contrary to the previous review, we excluded mental illness and HIV from our analysis. Contemporary maternal disability literature typically excludes psychiatric disabilities, focusing instead on physical, sensory, and intellectual and developmental disabilities (10), as perinatal mental health disorders represent the most common complication of childbearing with conditions that can emerge or be triggered by pregnancy itself rather than representing pre-existing disabilities (38). Similarly, HIV-related stigma and discrimination during pregnancy are shaped by context-specific cultural and social factors that vary across settings, and the intersection of HIV and maternal health has been extensively documented in the literature, including research on prevention of mother-to-child transmission, prenatal care access, social support, and communication (39–45). We also excluded drug therapy trials.

### Search strategy

In collaboration with the Biomedical Libraries at Dartmouth College, we developed a search strategy using the following databases for published articles from January 2012 to August 2025: Medline, APA PsycINFO, CINAHL Complete, Cochrane Library, Dissertations and Theses Global, Sociological Abstracts, Scopus, and Web of Science Social Science Citation Index. Two librarians developed and ran the search strategy. No limits were applied to the database searches. We compiled a list of exploded MeSH terms and keywords based on the initial search used by Malouf et al. to generate the following themes for our search: disabilities, maternal health services, and pregnancy care. We then utilized the Boolean operator “AND” to find the intersections between these themes. The study was registered with PROSPERO (CRD42023468318). The complete search strategy is provided in Supplementary Appendix A2.

### Study selection

All duplicates were removed using automated software features in Endnote and Rayyan. The first review was completed using *Rayyan* (46). To ensure rigor and minimize bias, six trained reviewers worked in three independent teams of two, with each reviewer blinded to their partner's assessments during initial screening. Each reviewer assessed articles for initial eligibility based on titles and abstracts. Once all reviewers had finished assessing their assigned articles, the review was unblinded, and each team met to resolve any conflicts. To be included in the first review, the title or abstract of the article must have shown that the literature (1) assessed an intervention (2) among women with disabilities (3) who were pregnant.

## Results

Our systematic review revealed a critical gap in the literature: no studies met our inclusion criteria among the 22,719 publications screened. While we found multiple studies that evaluated the safety and efficacy of medications to manage disability-associated conditions during pregnancy, none of these studies focused on the potential disabling impact of the conditions or the experience of pregnancy or childbirth for women living with disabilities, our intended focus. However, despite the systematic review identifying no interventions meeting our criteria, three recent pilot studies offer encouraging evidence that the field is advancing. These innovative projects are discussed in the next section.

## Discussion

While we found no interventions in the literature that were designed to improve pregnancy and childbirth experiences and related outcomes for women with disabilities, we identified three pilots that share a commitment to meaningful involvement of women with disabilities in maternal health research design and care delivery. This reflects growing recognition that “nothing about us, without us,” a core principle of the disability rights movement, must extend to research methodology (47).

The Together Project at the University of Surrey has developed and tested evidence-based resources, the “Together Toolkit” and “Maternity Passport”, to support good maternity care for people with learning disabilities (48). This work represents methodological progress by employing co-production principles, directly involving those with learning disabilities, researchers, and health and social care professionals in developing materials for patient education, provider training, and evaluation. Co-production of health represents a shift from viewing health as a product generated solely by professionals to a service created through the collaborative work of users and professionals (49). Engaging users (or patients) in the design of healthcare interventions and their delivery through consultations and iterative feedback can build trust and improve the adoption of new interventions, especially for patients such as those with disabilities, who have felt historically marginalized in the healthcare system (31, 50). In this project, midwives and parents found the toolkit and passport positively impacted maternity care through improvements in (1) learning disability awareness among providers, (2) accommodations and accessibility, (3) attitudes toward those with disabilities, (4) accessibility and appropriateness of health information and communication, and (5) trust. These co-produced implementation factors at both individual and system levels provide practical insights for scaling interventions to other groups.

The same research team developed learning disability awareness training for undergraduate midwifery students, co-produced and co-delivered with individuals with learning disabilities (51). Co-producing and co-delivering resources and education with people with lived experience led to an innovative intervention and positive experiences for those with learning disabilities. Rather than focusing solely on managing medical conditions, interventions that engage people with disabilities address persistent barriers related to provider knowledge and attitudes, healthcare system processes, and patient empowerment. This represents a shift toward the social model of disability, recognizing that barriers exist in environments and systems rather than with individuals (52).

The Accessible Pregnancy Action Plan (APAP), developed by Dr. Kara Ayers at Cincinnati Children's Hospital Medical Center, represents another innovative approach to empowering pregnant individuals with disabilities (53). The APAP utilizes health empowerment theory to support disabled pregnant people in identifying their priorities at various points in pregnancy, birth, and just after birth, working with a peer facilitator who is also a parent with a disability. This intervention moves beyond medical management to focus on patient-defined positive outcomes. The use of peer support reflects disability community values of shared experience and mutual aid.

### Strengths and limitations

Our study had notable strengths. We conducted a comprehensive search of the academic literature using both general disability terms and specific conditions associated with disability, ensuring broad capture of relevant interventions. A team of six reviewers independently screened over 20,000 studies, providing confidence in the thoroughness and reliability of our findings. However, our study also had several limitations. First, general accessibility accommodations such as wheelchair ramps or braille signage are designed to make buildings more accessible but would not appear in the health literature as pregnancy or childbirth interventions, and thus would be excluded from our review. Second, innovative models of care for women with disabilities, such as the Accessible Care Pregnancy Clinic in Canada (54), may not be captured in academic health literature despite potentially offering valuable disability-focused services. While a comprehensive web search of gray literature could identify such programs, this approach was beyond the scope of our systematic review. Third, our review did not include policy analysis, meaning that legislative changes with broad implications for people with disabilities, such as Section 504 of the U.S. Rehabilitation Act of 1973, which mandates accessibility in medical treatment (55), were not systematically examined. Although policies are not interventions *per se*, they represent important levers for resource allocation and healthcare system change, and policy advocacy efforts could serve as crucial targets for improving maternity care for women with disabilities.

## Conclusion

The emergence of promising interventions suggests growing recognition within the research community that disability-inclusive reproductive health research is both necessary and feasible. Additional gaps remain and must be addressed in future research. First, the identified interventions focus primarily on learning disabilities and broadly defined disabilities. Given the range of disability types, clinicians and researchers should assess whether an intervention addresses barriers that may be unique to the needs of women with physical disabilities, sensory impairments, or chronic conditions during pregnancy, as they will face distinct challenges. However, we report pilot interventions that highlight methodological approaches that can effectively accommodate the heterogeneity of disabilities while still yielding rigorous evidence, if developed in partnership with individuals who have lived experience. Second, traditional randomized controlled trials may not be the optimal approach for interventions that require a high degree of individualization. Observational studies emerging from clinical interventions, such as an accessible clinic for pregnant women with disabilities, can serve as a model for the delivery of tailored obstetric services. Robust outcome data demonstrating improved pregnancy and birth outcomes, including reproductive autonomy and quality of care experiences, alongside clinical pregnancy and birth outcomes, can demonstrate success. Third, intersectional approaches that consider race, class, sexuality, and other identities can help address unique reproductive health needs (56, 57). Evidence based practices are needed for intersecting identities as well. Research funding agencies must prioritize this area, and practitioners and researchers must collaborate to support community-engaged research with disabled communities.
