# Supplementary material for: A lack of evidence for disability-inclusive maternal health interventions and promising progress: an updated systematic review
Source: Front Glob Womens Health. 2025 Dec 16;6:1711871. doi: 10.3389/fgwh.2025.1711871 (PMC12748227; doi:10.3389/fgwh.2025.1711871)
Supplement: Supplementary file 1 [file Datasheet1.pdf]

Appendix 1. Conditions used in defining disability for the review

Disability conditions from Brown et al.

Category	Condition name
**PHYSICAL DISABILITIES**	
Congenital anomalies	* Congenital deformities of the spine (e.g., congenital scoliosis)
	Congenital deformities of the feet (e.g., club foot)
	Congenital musculoskeletal deformities of the chest (e.g., congenital funnel chest)
	Dwarfism, not elsewhere classified
	Hypopituitarism (e.g., pituitary dwarfism)
	Other congenital anomalies of the nervous system (e.g., congenital hydrocephalus)
	Other congenital musculoskeletal deformities (e.g osteochondroplasia)
	Reduction defects of lower limb
	* Reduction defects of unspecified limb (e.g., phocomelia NOS)
	Reduction defects of upper limb
	Spina bifida
	Syndactyly
Musculoskeletal disorders	Acromegaly and gigantism
	Ankylosing spondylitis
	* Chronic osteomyelitis
	Disc disorders
	Internal derangement of the knee
	Osteoarthritis
	Osteochondropathies
	* Osteonecrosis
	* Osteoporosis with history of pathological fracture
	Polymyalgia rheumatica
	Rheumatoid arthritis
	Spondylosis
Neurological disorders	Cerebral palsy
	Disorders of autonomic nervous system (e.g., idiopathic peripheral autonomic neuropathy)
	Epilepsy
	Hemiplegia
	Hereditary and idiopathic neuropathy
	Hereditary ataxia and other specified degenerative disorders of the nervous system classified elsewhere (e.g., Huntington's disease)
	* Mononeuropathies of the lower limb
	Multiple sclerosis
	Muscular dystrophy
	Myasthenia gravis
	Nerve root and plexus disorders
	Other demyelinating diseases of central nervous system (e.g., diffuse sclerosis)
	Other disorders of spinal cord (e.g., syringomyelia)
	Other extrapyramidal and movement disorders (e.g., essential tremor)
	Other paralytic syndromes (e.g., paraplegia)
	Other polyneuropathies (e.g., Guillain-Barre syndrome)
	Other specified degenerative diseases of the nervous system (e.g.,

	cerebral ataxia)
	Parkinson's disease
	Sequelae of cardiovascular disease
	Sequelae of poliomyelitis
	Spinal muscular atrophy and related syndromes (e.g., ALS)
Permanent injuries	* Brain injury
	Crushing injury of the lower limb
	Dependence on a wheelchair
	Dependence on other enabling machines and devices
	* Fracture of the lower back or pelvis
	Fracture of the vertebral column with spinal cord injury
	Other acquired deformities of limbs
	Traumatic amputation of the lower limb
	Traumatic amputation of the upper limb

SENSORY DISABILITIES

Hearing impairments	Conductive and sensorineural hearing loss
	Congenital malformations of ear causing impairment of hearing
Vision impairments	Blindness and low vision
	Cataracts
	Chorioretinal inflammation
	* Congenital malformations of the eye
	Disorders of globe (e.g., hypotony of eye)
	Disorders of the iris and ciliary body (e.g., chronic iridocyclitis)
	Disorders of visual cortex
	Glaucoma
	* Nystagmus and other irregular eye movements
	Other retinal disorders (e.g., other proliferative retinopathy)

INTELLECTUAL AND DEVELOPMENTAL DISABILITIES

	Autism spectrum disorder
	Fetal alcohol spectrum disorder
	Intellectual disability
	Intellectual disability resulting from chromosomal anomalies
	Other intellectual disabilities (e.g., fetal alcohol syndrome, tuberous sclerosis)
