# Supplementary material for: A lack of evidence for disability-inclusive maternal health interventions and promising progress: an updated systematic review
Source: Front Glob Womens Health. 2025 Dec 16;6:1711871. doi: 10.3389/fgwh.2025.1711871 (PMC12748227; doi:10.3389/fgwh.2025.1711871)
Supplement: Supplementary file 2 [file Datasheet2.pdf]

Appendix 2: Search Summary and Strategies

**Short Topic**	Interventions for disabilities in pregnant persons
**Librarians**	Search development: Pamela J. Bagley, MSLIS, PhD<br>Peer review: Heather B. Blunt, MSLIS
**Principal Investigator**	Alka Dev

Search Summary

Database	Platform	Years covered	Date conducted	# results
Medline All	Ovid	2012 - current	August 28, 2025	11750
APA PsycInfo	EBSCO	2012 - current	August 28, 2025	2865
CINAHL Complete	EBSCO	2012 - current	August 28, 2025	7271
Cochrane Library: Trials	Wiley	Cochrane Central Register of Controlled Trials Issue 7 of 12, July 2025	August 28, 2025	8575
Dissertations and Theses Global	ProQuest	2012 - current	August 28, 2025	620
Sociological Abstracts	ProQuest	2012 - current	August 28, 2025	797
Scopus	Elsevier	2012 - current	August 28, 2025	7895
Web of Science SSCI	Clarivate Analytics	2012 - current	August 28, 2025	1981
Total				41754
With duplicates removed				22719

Search Strategies

Medline All (Ovid)

Database(s): **Ovid MEDLINE(R) ALL** 1946 to August 28, 2025

#	Searches	Results
1	Maternal Health Services/ or *perinatal care/ or *postnatal care/ or *preconception care/ or *prenatal care/ or Pregnant People/ or exp Pregnancy/ or (pregnant or pregnancy).ti,ab. or ((maternal health or prenatal or pre-natal or prepartum or pre-partum or antenatal or ante-natal or perinatal or postnatal or post-natal or postpartum or post-partum or pueprium or puepral) adj3 (care or service* or healthcare)).ti,ab.	1216415
2	Persons with Disabilities/ or amputees/ or mobility limitation/ or exp Spinal Cord Injuries/ or paralysis/ or paraplegia/ or quadriplegia/ or Hemiplegia/ or exp Spinal Dysraphism/ or Cerebral Palsy/ or Arthritis, rheumatoid/ or exp Back Pain/ or Vision disorders/ or exp Blindness/ or Hearing disorders/ or Hearing loss/ or exp Deafness/ or Persons With Hearing Impairments/ or Persons with Visual Disabilities/ or Visually impaired persons/ or Mentally Ill Persons/ or Mental Disorders/ or exp Anxiety Disorders/ or Depressive disorder, major/ or exp "Schizophrenia Spectrum and Other Psychotic Disorders"/ or exp "Feeding and Eating Disorders"/ or exp Multiple Sclerosis/ or exp Muscular Dystrophies/ or exp Peripheral Nervous System Diseases/ or exp Stroke/ or exp Epilepsy/ or exp Migraine Disorders/ or exp Learning Disabilities/ or Intellectual Disability/ or Down syndrome/ or Persons with Intellectual Disabilities/ or Persons with Psychiatric Disorders/ or (disabled or disability).ti. or ((disabled or disability) adj5 (woman or women or female*)).ti,ab. or (spinal cord injur* or paralysis or paralyzed or paraplegi* or tetraplegi* or quadraplegi*).ti,ab. or ((physical or physically) adj (disabilit* or disabled)).ti. or (spina bifida or cerebral palsy or arthritis or (back adj (pain or ache))).ti,ab. or (blind or blindness or visually impaired or visual impairment or vision disorder*).ti,ab. or (deaf or deafness or hearing impair* or hearing loss).ti,ab. or (sensory disabil* or sensory impairment).ti,ab. or ((serious or sever* or major or chronic or longterm or long-term or persistent) adj3 (mentally ill or mental illness or mental disorder* or mental health)).ti,ab. or (manic* adj depress*).ti,ab. or ((serious or sever* or major or chronic or longterm or long-term or persistent) adj3 depress*).ti,ab. or ((bipolar or bi-polar) adj3 (depress* or disorder*)).ti,ab. or (schizophrenia or schizophrenic or psychos?s or psychotic).ti,ab. or (eating disorder* or disordered eating or anorexia nervosa or bulimia or binge eating).ti,ab. or (multiple sclerosis or muscular dystroph*).ti,ab. or peripheral neuropath*.ti,ab. or stroke.ti,ab. or (epilepsy or epileptic).ti,ab. or (migraine* or chronic headache*).ti,ab. or ((learning or intellectual) adj3 (disabilit* or handicap*)).ti,ab. or (mental retardation or mental disabilit*).ti,ab.	2674662

3	Pilot projects/ or (randomized controlled trial or controlled clinical trial or multicenter study).pt. or (randomized or randomised or placebo or randomly or trial or groups).ab. or intervention*.ti. or (intervention* adj6 (clinician* or collaborat* or community or complex or design* or doctor* or educational or family doctor* or financial or GP or general practice* or hospital* or impact* or improv* or individuali?e* or individuali?ing or interdisciplin* or multicomponent or multi-component or multidisciplin* or multi-disciplin* or multifacet* or multi-facet* or multimodal* or multi-modal* or personali?e* or personali?ing or pharmacies or pharmacist* or pharmacy or physician* or practitioner* or prescrib* or prescription* or primary care or professional* or provider* or regulatory or tailor* or target* or team* or usual care)).ab. or (collaborativ* or collaboration* or tailored or personali?ed).ti,ab. or ((exp hospitals/ or exp Hospitalization/ or exp Patients/ or exp Nurses/ or exp Nursing/) and (study.ti. or evaluation studies as topic/)) or demonstration project*.ti,ab. or ("pre test*" or pretest* or posttest* or "post test*" or (pre adj5 post)).ti,ab. or (pre-workshop or post-workshop or (before adj3 workshop) or (after adj3 workshop)).ti,ab. or ((study adj3 aim?) or "our study").ab. or (before adj10 (after or during)).ti,ab. or ("quasi-experiment*" or quasiexperiment* or "quasi random*" or quasirandom* or "quasi control*" or quasicontrol* or ((quasi* or experimental) adj3 (method* or study or trial or design*))).ti,ab,hw. or ("time series" adj2 interrupt*).ti,ab,hw. or (time points adj3 (over or multiple or three or four or five or six or seven or eight or nine or ten or eleven or twelve or month* or hour? or day? or "more than")).ab. or pilot.ti. or (multicentre or multicenter or multi-centre or multi-center).ti. or random*.ti,ab. or controlled.ti. or (control adj3 (area or cohort? or compar? or condition or group? or intervention? or participant? or study)).ab.	7644697
4	1 and 2 and 3	20590
5	limit 4 to yr="2012 -Current"	11750

APA PsycInfo (Ebsco)

S5	S3 AND S2 AND S1<br>Limit to 01/01/2012 - 12/12/2025	2,865
S4	S3 AND S2 AND S1	4,357
S3	DE "Randomized Controlled Trials" OR DE "Clinical Trials" OR ((DE "Hospitals" OR DE "Hospitalization" OR DE "Patients" OR DE "Hospitalized Patients" OR DE "Medical Patients" OR DE "Nurses") AND ((TI study)) OR XB (intervention* or collaborativ* or collaboration* or tailored or personalized or personalised or “demonstration project*” or "pre test*" or pretest* or posttest* or "post test*" or (pre N5 post) or pre-workshop or post-workshop or (before N3 workshop) or (after N3 workshop) or (before N10 (after or during)) or "quasi-	1,120,031

	experiment*" or quasiexperiment* or "quasi random*" or quasirandom* or "quasi control*" or quasicontrol* or ((quasi* or experimental) N3 (method* or study or trial or design*)) or ("time series" N2 interrupt*) or pilot or multicentre or multicenter or multi-centre or multi-center or random* or controlled)	
S2	DE "Disabilities" OR DE "Disability Management" OR DE "Learning Disabilities" OR DE "Multiple Disabilities" OR DE "Reading Disabilities" OR DE "Learning Disorders" OR DE "Dyslexia" OR DE "Accessibility (Disabilities)" OR DE "Deaf Blind" OR DE "Blindness" OR DE "Deafness" OR DE "Multiple Disabilities" OR DE "Bipolar Disorder" OR DE "Bipolar I Disorder" OR DE "Bipolar II Disorder" OR DE "Cyclothymic Disorder" OR DE "Mania" OR DE "Hypomania" OR DE "Affective Psychosis" OR DE "Major Depression" OR DE "Treatment Resistant Depression" OR DE "Anxiety Disorders" OR DE "Mental Disorders" OR DE "Generalized Anxiety Disorder" OR DE "Eating Disorders" OR DE "Anorexia Nervosa" OR DE "Avoidant/Restrictive Food Intake Disorder" OR DE "Binge Eating Disorder" OR DE "Bulimia" OR DE "Feeding Disorders" OR DE "Hyperphagia" OR DE "Kleine Levin Syndrome" OR DE "Orthorexia" OR DE "Pica" OR DE "Purging (Eating Disorders)" OR DE "Rumination (Eating)" OR DE "Multiple Sclerosis" OR DE "Muscular Dystrophy" OR DE "Cerebrovascular Accidents" OR DE "Epilepsy" OR DE "Epileptic Seizures" OR DE "Migraine Headache" OR DE "Developmental Disabilities" OR DE "Back Pain" OR DE "Paralysis" OR DE "Cerebral Palsy" OR DE "Hemiparesis" OR DE "Hemiplegia" OR DE "Paraplegia" OR DE "Quadriplegia" OR DE "Rheumatoid Arthritis" OR XB (disabled or disability or ((physical or physically) N1 (disabilit* or disabled)) or ((disabled or disability) N5 (woman or women or female*)) or “spinal cord injur*” or paralysis or paralyzed or paraplegi* or tetraplegi* or quadraplegi* or hemiplegi* or “spina bifida” or “cerebral palsy” or arthritis or (back N1 (pain or ache)) or blind or blindness or “visually impaired” or “visual impairment” or “vision disorder*” or deaf or deafness or “hearing impair*” or “hearing loss” or “sensory disabil*” or ”sensory impairment” or ((serious or sever* or major or chronic or longterm or long-term or persistent) N3 (“mentally ill” or “mental illness” or “mental disorder*” or “mental health”)) or (manic* N1 depress*) or ((serious or sever* or major or chronic or longterm or long-term or persistent) N3 depress*) or ((bipolar or bi-polar) N3 (depress* or disorder*)) or schizophrenia or schizophrenic or psychosis or psychoses or psychotic or “eating disorder*” or “disordered eating” or “anorexia nervosa” or bulimia or “binge eating” or “multiple sclerosis” or “muscular dystroph*” or “ peripheral neuropath*” or stroke or epilepsy or epileptic or migraine* or “chronic headache*” or ((learning or intellectual) N3 (disabilit* or handicap*)) or “mental retardation” or “mental disabilit*”)	966,148
S1	DE "Prenatal Care" OR DE "Childbirth Training" OR DE "Pregnancy" OR DE "Adolescent Pregnancy" OR DE "Expectant Mothers" OR XB (pregnant or pregnancy or (("maternal health" or prenatal or pre-natal or prepartum or pre-	81,787

	partum or antenatal or ante-natal or perinatal or postnatal or post-natal or postpartum or post-partum or pueprium or puepral) N3 (care or service* or healthcare)))	

CINAHL Complete (Ebsco)

S5	S3 AND S2 AND S1<br>Limit to 01/01/2012 - 12/12/2025	7,271
S4	S3 AND S2 AND S1	10,185
S3	MH "Randomized Controlled Trials" OR MH "Clinical Trials" OR MH "Multicenter Studies" OR MH "Pilot Studies" OR ((MH "Hospitals+" OR MH "Hospitalization" OR MH "Patients+" OR MH "Nurses+" OR "Nursing as a Profession+" OR MH "Research, Nursing") AND (TI study OR MH "Evaluation Research+")) OR XB (intervention* or collaborativ* or collaboration* or tailored or personalized or personalised or "demonstration project*" or "pre test*" or pretest* or posttest* or "post test*" or (pre N5 post) or pre-workshop or post-workshop or (before N3 workshop) or (after N3 workshop) or (before N10 (after or during)) or "quasi-experiment*" or quasiexperiment* or "quasi random*" or quasirandom* or "quasi control*" or quasicontrol* or ((quasi* or experimental) N3 (method* or study or trial or design*)) or ("time series" N2 interrupt*) or pilot or multicentre or multicenter or multi-centre or multi-center or random* or controlled)	1,765,030
S2	MH "Persons with Disabilities" OR MH "Persons with Hearing Disabilities" OR MH "Persons with Intellectual Disabilities" OR MH "Persons with Mental Disorders" OR MH "Persons with Visual Disabilities" OR MH "Amputees" OR MH "Persons with Intellectual Disabilities" OR MH "Spinal Cord Injuries+" OR MH "Paralysis" OR (MH "Hemiplegia" OR MH "Paraplegia+" OR MH "Quadriplegia+" OR MH "Spina Bifida" OR MH "Cerebral Palsy" OR MH "Arthritis, Rheumatoid" OR MH "Back Pain+" OR MH "Vision Disorders" OR MH "Blindness+" OR MH "Hearing Disorders+" OR MH "Mental Disorders" OR MH "Anxiety Disorders+" OR MH "Affective Disorders+" OR MH "Depression" OR MH "Psychotic Disorders+" OR MH "Eating Disorders+" OR (MH "Multiple Sclerosis+" OR MH "Muscular Dystrophy+" OR MH "Peripheral Nervous System Diseases" OR MH "Stroke+" OR MH "Epilepsy+" OR MH "Migraine" OR MH "Learning Disorders+" OR MH "Intellectual Disability+" OR XB (disabled or disability or ((physical or physically) N1 (disabilit* or disabled)) or ((disabled or disability) N5 (woman or women or female*)) or "spinal cord injur*" or paralysis or paralyzed or paraplegi* or tetraplegi* or quadraplegi* or hemiplegi* or "spina bifida" or "cerebral palsy" or arthritis or (back N1 (pain or ache)) or blind or blindness or "visually impaired" or "visual impairment" or "vision disorder*" or deaf or deafness or "hearing impair*" or "hearing loss" or "sensory disabil*" or "sensory impairment" or ((serious or sever* or major or	1,093,180

	chronic or longterm or long-term or persistent) N3 (“mentally ill” or “mental illness” or “mental disorder*” or “mental health”)) or (manic* N1 depress*) or ((serious or sever* or major or chronic or longterm or long-term or persistent) N3 depress*) or ((bipolar or bi-polar) N3 (depress* or disorder*)) or schizophrenia or schizophrenic or psychosis or psychoses or psychotic or “eating disorder*” or “disordered eating” or “anorexia nervosa” or bulimia or “binge eating” or “multiple sclerosis” or “muscular dystroph*” or “ peripheral neuropath*” or stroke or epilepsy or epileptic or migraine* or “chronic headache*” or ((learning or intellectual) N3 (disabilit* or handicap*)) or “mental retardation” or “mental disabilit*”)	
S1	MH "Maternal Health Services" OR MH "Postnatal Care" OR MM "Prenatal Care" OR MM "Prepregnancy Care" OR MH "Expectant Mothers" OR MH "Perinatal Care" OR MH "Labor Support" OR MH "Management of Labor" OR MH "Intrapartum Care" OR MH "Pregnancy+" OR XB (pregnant or pregnancy or (("maternal health" or prenatal or pre-natal or prepartum or pre-partum or antenatal or ante-natal or perinatal or postnatal or post-natal or postpartum or post-partum or pueprium or puepral) N3 (care or service* or healthcare)))	331,360

Cochrane Central Register of Controlled Trials (Wiley)

#1	(((pregnant or pregnancy or (("maternal health" or prenatal or pre-natal or prepartum or pre-partum or antenatal or ante-natal or perinatal or postnatal or post-natal or postpartum or post-partum or pueprium or puepral) Near/3 (care or service* or healthcare))))):ti OR (((pregnant or pregnancy or (("maternal health" or prenatal or pre-natal or prepartum or pre-partum or antenatal or ante-natal or perinatal or postnatal or post-natal or postpartum or post-partum or pueprium or puepral) Near/3 (care or service* or healthcare))))):ab	76125
#2	(((disabled or disability or ((physical or physically) Near/1 (disabilit* or disabled)) or ((disabled or disability) Near/5 (woman or women or female*)) or (“spinal cord” NEXT injur*) or paralysis or paralyzed or paraplegi* or tetraplegi* or quadraplegi* or hemiplegi* or “spina bifida” or “cerebral palsy” or arthritis or (back Near/1 (pain or ache)) or blind or blindness or “visually impaired” or “visual impairment” or (vision NEXT disorder*) or deaf or deafness or (hearing NEXT impair*) or “hearing loss” or (sensory NEXT disabil*) or ”sensory impairment” or ((serious or sever* or major or chronic or longterm or long-term or persistent) Near/3 (“mentally ill” or “mental illness” or (mental NEXT disorder*) or “mental health”)) or (manic* Near/1 depress*) or ((serious or sever* or major or chronic or longterm or long-term or persistent) Near/3 depress*) or ((bipolar or bi-polar) Near/3 (depress* or disorder*)) or schizophrenia or schizophrenic or psychosis or psychoses or	506774

	psychotic or (eating NEXT disorder*) or “disordered eating” or “anorexia nervosa” or bulimia or “binge eating” or “multiple sclerosis” or (muscular NEXT dystroph*) or (peripheral NEXT neuropath*) or stroke or epilepsy or epileptic or migraine* or (chronic NEXT headache*) or ((learning or intellectual) Near/3 (disabilit* or handicap*)) or “mental retardation” or (mental NEXT disabilit*)))):ti<br>OR<br>((((disabled or disability) Near/5 (woman or women or female*)) or (spinal cord NEXT injur*) or paralysis or paralyzed or paraplegi* or tetraplegi* or quadraplegi* or hemiplegi* or “spina bifida” or “cerebral palsy” or arthritis or (back Near/1 (pain or ache)) or blind or blindness or “visually impaired” or “visual impairment” or (vision NEXT disorder*) or deaf or deafness or (hearing NEXT impair*) or “hearing loss” or (sensory NEXT disabil*) or ”sensory impairment” or ((serious or sever* or major or chronic or longterm or long-term or persistent) Near/3 (“mentally ill” or “mental illness” or (mental NEXT disorder*) or “mental health”)) or (manic* Near/1 depress*) or ((serious or sever* or major or chronic or longterm or long-term or persistent) Near/3 depress*) or ((bipolar or bi-polar) Near/3 (depress* or disorder*)) or schizophrenia or schizophrenic or psychosis or psychoses or psychotic or (eating NEXT disorder*) or “disordered eating” or “anorexia nervosa” or bulimia or “binge eating” or “multiple sclerosis” or (muscular NEXT dystroph*) or (peripheral NEXT neuropath*) or stroke or epilepsy or epileptic or migraine* or (chronic NEXT headache*) or ((learning or intellectual) Near/3 (disabilit* or handicap*)) or “mental retardation” or (mental NEXT disabilit*))):ab	
#3	#1 AND #2<br>Limits: with Publication Year from 2012 to 2025, in Trials	8575

Dissertations and Theses Global (ProQuest)

Set	Search	Results
S5	[S1] AND [S2] AND [S3]<br>Limits applied 2012-01-01 - 2025-08-28	620
S4	[S1] AND [S2] AND [S3]	1089
S3	title(intervention* or collaborativ* or collaboration* or tailored or personalized or personalised or “demonstration project*” or "pre test*" or pretest* or posttest* or "post test*" or ("pre" N/5 post) or pre-workshop or post-workshop or (before N/3 workshop) or (after N/3 workshop) or (before N/10 (after or during)) or "quasi-experiment*" or quasiexperiment* or "quasi random*" or quasirandom* or "quasi control*" or quasicontrol* or	1,478,056

	((quasi* or experimental) N/3 (method* or study or trial or design*)) or ("time series" N/2 interrupt*) or pilot or multicentre or multicenter or multi-centre or multi-center or random* or controlled) OR abstract(randomized or randomised or placebo or randomly or trial or groups or (intervention* N/6 (clinician* or collaborat* or community or complex or design* or doctor* or educational or "family doctor*" or financial or GP or "general practice*" or hospital* or impact* or improv* or individualize* or individualise* or individualizing or individualising or interdisciplin* or multicomponent or multi-component or multidisciplin* or multi-disciplin* or multifacet* or multi-facet* or multimodal* or multi-modal* or personalize* or personalise* or personalizing or personalising or pharmacies or pharmacist* or pharmacy or physician* or practitioner* or prescrib* or prescription* or "primary care" or professional* or provider* or regulatory or tailor* or target* or team* or "usual care")) or collaborativ* or collaboration* or tailored or "demonstration project*" or "pre test*" or pretest* or posttest* or "post test*" or ("pre" N/5 post) or pre-workshop or post-workshop or (before N/3 workshop) or (after N/3 workshop) or (study N/3 (aim or aims)) or "our study" or (before N/10 (after or during)) or "quasi-experiment*" or quasiexperiment* or "quasi random*" or quasirandom* or "quasi control*" or quasicontrol* or ((quasi* or experimental) N/3 (method* or study or trial or design*)) or ("time series" N/2 interrupt*) or ("time points" N/3 (over or multiple or three or four or five or six or seven or eight or nine or ten or eleven or twelve or month* or hour or hours or day or days or "more than")) or random* or (control N/3 (area or cohort or cohorts or compare or condition or group or groups or intervention or interventions or participant or participants or study)))	
S2	title((disabled or disability or ((physical or physically) N/1 (disabilit* or disabled)) or ((disabled or disability) N/5 (woman or women or female*)) or "spinal cord injur*" or paralysis or paralyzed or paraplegi* or tetraplegi* or quadraplegi* or hemiplegi* or "spina bifida" or "cerebral palsy" or arthritis or (back N/1 (pain or ache)) or blind or blindness or "visually impaired" or "visual impairment" or "vision disorder*" or deaf or deafness or "hearing impair*" or "hearing loss" or "sensory disabil*" or "sensory impairment" or ((serious or sever* or major or chronic or longterm or long-term or persistent) N/3 ("mentally ill" or "mental illness" or "mental disorder*" or "mental health")) or (manic* N/1 depress*) or ((serious or sever* or major or chronic or longterm or long-term or persistent) N3 depress*) or ((bipolar or bi-polar) N/3 (depress* or disorder*)) or schizophrenia or schizophrenic or psychosis or psychoses or psychotic or "eating disorder*" or "disordered eating" or "anorexia nervosa" or bulimia or "binge eating" or "multiple sclerosis" or "muscular dystroph*" or " peripheral neuropath*" or stroke or epilepsy or epileptic or migraine* or "chronic headache*" or ((learning or intellectual) N/3 (disabilit* or handicap*)) or "mental retardation" or	155,772

	“mental disabilit*”)) OR abstract(((disabled or disability) N/5 (woman or women or female*)) or “spinal cord injur*” or paralysis or paralyzed or paraplegi* or tetraplegi* or quadraplegi* or hemiplegi* or “spina bifida” or “cerebral palsy” or arthritis or (back N/1 (pain or ache)) or blind or blindness or “visually impaired” or “visual impairment” or “vision disorder*” or deaf or deafness or “hearing impair*” or “hearing loss” or “sensory disabil*” or ”sensory impairment” or ((serious or sever* or major or chronic or longterm or long-term or persistent) N/3 (“mentally ill” or “mental illness” or “mental disorder*” or “mental health”)) or (manic* N/1 depress*) or ((serious or sever* or major or chronic or longterm or long-term or persistent) N/3 depress*) or ((bipolar or bi-polar) N/3 (depress* or disorder*)) or schizophrenia or schizophrenic or psychosis or psychoses or psychotic or “eating disorder*” or “disordered eating” or “anorexia nervosa” or bulimia or “binge eating” or “multiple sclerosis” or “muscular dystroph*” or “ peripheral neuropath*” or stroke or epilepsy or epileptic or migraine* or “chronic headache*” or ((learning or intellectual) N/3 (disabilit* or handicap*)) or “mental retardation” or “mental disabilit*”)	
S1	title((pregnant or pregnancy or (("maternal health" or prenatal or pre-natal or prepartum or pre-partum or antenatal or ante-natal or perinatal or postnatal or post-natal or postpartum or post-partum or pueprium or puepral) N/3 (care or service* or healthcare)))) OR abstract((pregnant or pregnancy or (("maternal health" or prenatal or pre-natal or prepartum or pre-partum or antenatal or ante-natal or perinatal or postnatal or post-natal or postpartum or post-partum or pueprium or puepral) N/3 (care or service* or healthcare))))	39,496

Sociological Abstracts (ProQuest)

Set	Search	Results
**S5**	[S1] AND [S2] AND [S3]<br>Limits applied<br>Databases: Sociological Abstracts<br>Narrowed by: Entered date: 2012-01-01 - 2025-08-28;<br>Exclude: Document type: Literature Review; Commentary	797
**S4**	[S1] AND [S2] AND [S3]	**1,002**

**S3**	MAINSUBJECT.EXACT("Clinical trials") OR title(intervention* or collaborativ* or collaboration* or tailored or personalized or personalised or “demonstration project*” or "pre test*" or pretest* or posttest* or "post test*" or ("pre" N/5 post) or pre-workshop or post-workshop or (before N/3 workshop) or (after N/3 workshop) or (before N/10 (after or during)) or "quasi-experiment*" or quasiexperiment* or "quasi random*" or quasirandom* or "quasi control*" or quasicontrol* or ((quasi* or experimental) N/3 (method* or study or trial or design*)) or ("time series" N/2 interrupt*) or pilot or multicentre or multicenter or multi-centre or multi-center or random* or controlled) OR abstract(randomized or randomised or placebo or randomly or trial or groups or (intervention* N/6 (clinician* or collaborat* or community or complex or design* or doctor* or educational or “family doctor*” or financial or GP or “general practice*” or hospital* or impact* or improv* or individualize* or individualise* or individualizing or individualising or interdisciplin* or multicomponent or multi-component or multidisciplin* or multi-disciplin* or multifacet* or multi-facet* or multimodal* or multi-modal* or personalize* or personalise* or personalizing or personalising or pharmacies or pharmacist* or pharmacy or physician* or practitioner* or prescrib* or prescription* or “primary care” or professional* or provider* or regulatory or tailor* or target* or team* or “usual care”)) or collaborativ* or collaboration* or tailored or “demonstration project*” or "pre test*" or pretest* or posttest* or "post test*" or ("pre" N/5 post) or pre-workshop or post-workshop or (before N/3 workshop) or (after N/3 workshop) or (study N/3 (aim or aims)) or "our study" or (before N/10 (after or during)) or "quasi-experiment*" or quasiexperiment* or "quasi random*" or quasirandom* or "quasi control*" or quasicontrol* or ((quasi* or experimental) N/3 (method* or study or trial or design*)) or ("time series" N/2 interrupt*) or (“time points” N/3 (over or multiple or three or four or five or six or seven or eight or nine or ten or eleven or twelve or month* or hour or hours or day or days or "more than")) or random* or (control N/3 (area or cohort or cohorts or compare or condition or group or groups or intervention or interventions or participant or participants or study)))	**484,075**
**S2**	MAINSUBJECT.EXACT.EXPLODE("Disability") OR MAINSUBJECT.EXACT.EXPLODE("Disabled people") OR MAINSUBJECT.EXACT("Epilepsy") OR MAINSUBJECT.EXACT("Down syndrome") OR MAINSUBJECT.EXACT("Blindness") OR MAINSUBJECT.EXACT("Cerebral palsy") OR	**106,587**

	MAINSUBJECT.EXACT.EXPLODE("Mental disorders") OR MAINSUBJECT.EXACT("Deafness") OR MAINSUBJECT.EXACT("Arthritis") OR MAINSUBJECT.EXACT("Visual impairment") OR title((disabled or disability or ((physical or physically) N/1 (disabilit* or disabled)) or ((disabled or disability) N/5 (woman or women or female*)) or “spinal cord injur*” or paralysis or paralyzed or paraplegi* or tetraplegi* or quadraplegi* or hemiplegi* or “spina bifida” or “cerebral palsy” or arthritis or (back N/1 (pain or ache)) or blind or blindness or “visually impaired” or “visual impairment” or “vision disorder*” or deaf or deafness or “hearing impair*” or “hearing loss” or “sensory disabil*” or ”sensory impairment” or ((serious or sever* or major or chronic or longterm or long-term or persistent) N/3 (“mentally ill” or “mental illness” or “mental disorder*” or “mental health”)) or (manic* N/1 depress*) or ((serious or sever* or major or chronic or longterm or long-term or persistent) N3 depress*) or ((bipolar or bi-polar) N/3 (depress* or disorder*)) or schizophrenia or schizophrenic or psychosis or psychoses or psychotic or “eating disorder*” or “disordered eating” or “anorexia nervosa” or bulimia or “binge eating” or “multiple sclerosis” or “muscular dystroph*” or “ peripheral neuropath*” or stroke or epilepsy or epileptic or migraine* or “chronic headache*” or ((learning or intellectual) N/3 (disabilit* or handicap*)) or “mental retardation” or “mental disabilit*”)) OR abstract(((disabled or disability) N/5 (woman or women or female*)) or “spinal cord injur*” or paralysis or paralyzed or paraplegi* or tetraplegi* or quadraplegi* or hemiplegi* or “spina bifida” or “cerebral palsy” or arthritis or (back N/1 (pain or ache)) or blind or blindness or “visually impaired” or “visual impairment” or “vision disorder*” or deaf or deafness or “hearing impair*” or “hearing loss” or “sensory disabil*” or ”sensory impairment” or ((serious or sever* or major or chronic or longterm or long-term or persistent) N/3 (“mentally ill” or “mental illness” or “mental disorder*” or “mental health”)) or (manic* N/1 depress*) or ((serious or sever* or major or chronic or longterm or long-term or persistent) N/3 depress*) or ((bipolar or bi-polar) N/3 (depress* or disorder*)) or schizophrenia or schizophrenic or psychosis or psychoses or psychotic or “eating disorder*” or “disordered eating” or “anorexia nervosa” or bulimia or “binge eating” or “multiple sclerosis” or “muscular dystroph*” or “ peripheral neuropath*” or stroke or epilepsy or epileptic or migraine* or “chronic headache*” or ((learning or intellectual) N/3 (disabilit* or handicap*)) or “mental retardation” or “mental disabilit*”)	

**S1**	MAINSUBJECT.EXACT("Prenatal care") OR MAINSUBJECT.EXACT.EXPLODE("Pregnancy") OR title((pregnant or pregnancy or (("maternal health" or prenatal or pre-natal or prepartum or pre-partum or antenatal or ante-natal or perinatal or postnatal or post-natal or postpartum or post-partum or pueprium or puepral) N/3 (care or service* or healthcare)))) OR abstract((pregnant or pregnancy or (("maternal health" or prenatal or pre-natal or prepartum or pre-partum or antenatal or ante-natal or perinatal or postnatal or post-natal or postpartum or post-partum or pueprium or puepral) N/3 (care or service* or healthcare))))	**25,260**

Scopus (Elsevier)

5	((TITLE (((pregnant OR pregnancy OR (("maternal health" OR prenatal OR pre-natal OR prepartum OR pre-partum OR antenatal OR ante-natal OR perinatal OR postnatal OR post-natal OR postpartum OR post-partum OR pueprium OR puepral) W/3 (care OR service* OR healthcare))))) OR ABS (((pregnant OR pregnancy OR (("maternal health" OR prenatal OR pre-natal OR prepartum OR pre-partum OR antenatal OR ante-natal OR perinatal OR postnatal OR post-natal OR postpartum OR post-partum OR pueprium OR puepral) W/3 (care OR service* OR healthcare)))))) AND (TITLE (disabled OR disability OR ((physical OR physically) W/1 (disabilit* OR disabled)) OR ((disabled OR disability) W/5 (woman OR women OR female*)) OR "spinal cord injur*" OR paralysis OR paralyzed OR paraplegi* OR tetraplegi* OR quadraplegi* OR hemiplegi* OR "spina bifida" OR "cerebral palsy" OR arthritis OR (back W/1 (pain OR ache)) OR blind OR blindness OR "visually impaired" OR "visual impairment" OR "vision disorder*" OR deaf OR deafness OR "hearing impair*" OR "hearing loss" OR "sensory disabil*" OR "sensory impairment" OR ((serious OR sever* OR major OR chronic OR longterm OR long-term OR persistent) W/3 ("mentally ill" OR "mental illness" OR "mental disorder*" OR "mental health")) OR (manic* W/1 depress*) OR ((serious OR sever* OR major OR chronic OR longterm OR long-term OR persistent) W/3 depress*) OR ((bipolar OR bi-polar) W/3 (depress* OR disorder*)) OR schizophrenia OR schizophrenic OR psychosis OR psychoses OR psychotic OR "eating disorder*" OR "disordered eating" OR "anorexia nervosa" OR bulimia OR "binge eating" OR "multiple sclerosis" OR "muscular dystroph*" OR " peripheral neuropath*" OR stroke OR epilepsy OR epileptic OR migraine* OR "chronic headache*" OR ((learning OR intellectual) W/3 (disabilit* OR handicap*)) OR "mental retardation" OR "mental disabilit*") OR ABS (((disabled OR disability) W/5 (woman OR women OR female*)) OR "spinal cord injur*" OR paralysis OR paralyzed OR paraplegi* OR tetraplegi* OR quadraplegi* OR hemiplegi* OR "spina bifida" OR "cerebral palsy" OR arthritis OR (back W/1 (pain OR ache)) OR blind OR blindness OR "visually impaired"	7,895 results

	OR "visual impairment" OR "vision disorder*" OR deaf OR deafness OR "hearing impair*" OR "hearing loss" OR "sensory disabil*" OR "sensory impairment" OR ((serious OR sever* OR major OR chronic OR longterm OR long-term OR persistent) W/3 ("mentally ill" OR "mental illness" OR "mental disorder*" OR "mental health")) OR (manic* W/1 depress*) OR ((serious OR sever* OR major OR chronic OR longterm OR long-term OR persistent) W/3 depress*) OR ((bipolar OR bi-polar) W/3 (depress* OR disorder*)) OR schizophrenia OR schizophrenic OR psychosis OR psychoses OR psychotic OR "eating disorder*" OR "disordered eating" OR "anorexia nervosa" OR bulimia OR "binge eating" OR "multiple sclerosis" OR "muscular dystroph*" OR " peripheral neuropath*" OR stroke OR epilepsy OR epileptic OR migraine* OR "chronic headache*" OR ((learning OR intellectual) W/3 (disabilit* OR handicap*)) OR "mental retardation" OR "mental disabilit*")) AND (TITLE (intervention* OR collaborativ* OR collaboration* OR tailored OR personalized OR personalised OR "demonstration project*" OR "pre test*" OR pretest* OR posttest* OR "post test*" OR (pre W/5 post) OR pre-workshop OR post-workshop OR (before W/3 workshop) OR (after W/3 workshop) OR (before W/10 (after OR during)) OR "quasi-experiment*" OR quasiexperiment* OR "quasi random*" OR quasirandom* OR "quasi control*" OR quasicontrol* OR ((quasi* OR experimental) W/3 (method* OR study OR trial OR design*)) OR ("time series" W/2 interrupt*) OR pilot OR multicentre OR multicenter OR multi-centre OR multi-center OR random* OR controlled) OR ABS (randomized OR randomised OR placebo OR randomly OR trial OR groups OR (intervention* W/6 (clinician* OR collaborat* OR community OR complex OR design* OR doctor* OR educational OR "family doctor*" OR financial OR GP OR "general practice*" OR hospital* OR impact* OR improv* OR individualize* OR individualise* OR individualizing OR individualising OR interdisciplin* OR multicomponent OR multi-component OR multidisciplin* OR multi-disciplin* OR multifacet* OR multi-facet* OR multimodal* OR multi-modal* OR personalize* OR personalise* OR personalizing OR personalising OR pharmacies OR pharmacist* OR pharmacy OR physician* OR practitioner* OR prescrib* OR prescription* OR "primary care" OR professional* OR provider* OR regulatory OR tailor* OR target* OR team* OR "usual care")) OR collaborativ* OR collaboration* OR tailored OR "demonstration project*" OR "pre test*" OR pretest* OR posttest* OR "post test*" OR (pre W/5 post) OR pre-workshop OR post-workshop OR (before W/3 workshop) OR (after W/3 workshop) OR (study W/3 (aim OR aims)) OR "our study" OR (before W/10 (after OR during)) OR "quasi-experiment*" OR quasiexperiment* OR "quasi random*" OR quasirandom* OR "quasi control*" OR quasicontrol* OR ((quasi* OR experimental) W/3 (method* OR study OR trial OR design*)) OR ("time series" W/2 interrupt*) OR ("time points" W/3 (over OR multiple OR three OR four OR five OR six OR seven OR eight OR nine OR ten OR eleven OR twelve OR month* OR hour OR hours OR day OR days OR "more than")) OR random* OR (control W/3 (area OR cohort OR cohorts OR compare OR condition OR group OR groups OR intervention OR interventions OR	

	participant OR participants OR study))))) AND (PUBYEAR > 2011) AND NOT ((INDEXTERMS (animals OR animal)) AND NOT (INDEXTERMS (humans OR human))) AND (EXCLUDE (DOCTYPE , "le") OR EXCLUDE (DOCTYPE , "re") OR EXCLUDE (DOCTYPE , "ed")) AND (LIMIT-TO (LANGUAGE , "English"))	
4	TITLE (((pregnant OR pregnancy OR (("maternal health" OR prenatal OR pre-natal OR prepartum OR pre-partum OR antenatal OR ante-natal OR perinatal OR postnatal OR post-natal OR postpartum OR post-partum OR pueprium OR puepral) W/3 (care OR service* OR healthcare))))) OR ABS (((pregnant OR pregnancy OR (("maternal health" OR prenatal OR pre-natal OR prepartum OR pre-partum OR antenatal OR ante-natal OR perinatal OR postnatal OR post-natal OR postpartum OR post-partum OR pueprium OR puepral) W/3 (care OR service* OR healthcare)))))) AND (TITLE (disabled OR disability OR ((physical OR physically) W/1 (disabilit* OR disabled)) OR ((disabled OR disability) W/5 (woman OR women OR female*)) OR "spinal cord injur*" OR paralysis OR paralyzed OR paraplegi* OR tetraplegi* OR quadraplegi* OR hemiplegi* OR "spina bifida" OR "cerebral palsy" OR arthritis OR (back W/1 (pain OR ache)) OR blind OR blindness OR "visually impaired" OR "visual impairment" OR "vision disorder*" OR deaf OR deafness OR "hearing impair*" OR "hearing loss" OR "sensory disabil*" OR "sensory impairment" OR ((serious OR sever* OR major OR chronic OR longterm OR long-term OR persistent) W/3 ("mentally ill" OR "mental illness" OR "mental disorder*" OR "mental health")) OR (manic* W/1 depress*) OR ((serious OR sever* OR major OR chronic OR longterm OR long-term OR persistent) W/3 depress*) OR ((bipolar OR bi-polar) W/3 (depress* OR disorder*)) OR schizophrenia OR schizophrenic OR psychosis OR psychoses OR psychotic OR "eating disorder*" OR "disordered eating" OR "anorexia nervosa" OR bulimia OR "binge eating" OR "multiple sclerosis" OR "muscular dystroph*" OR " peripheral neuropath*" OR stroke OR epilepsy OR epileptic OR migraine* OR "chronic headache*" OR ((learning OR intellectual) W/3 (disabilit* OR handicap*)) OR "mental retardation" OR "mental disabilit*") OR ABS (((disabled OR disability) W/5 (woman OR women OR female*)) OR "spinal cord injur*" OR paralysis OR paralyzed OR paraplegi* OR tetraplegi* OR quadraplegi* OR hemiplegi* OR "spina bifida" OR "cerebral palsy" OR arthritis OR (back W/1 (pain OR ache)) OR blind OR blindness OR "visually impaired" OR "visual impairment" OR "vision disorder*" OR deaf OR deafness OR "hearing impair*" OR "hearing loss" OR "sensory disabil*" OR "sensory impairment" OR ((serious OR sever* OR major OR chronic OR longterm OR long-term OR persistent) W/3 ("mentally ill" OR "mental illness" OR "mental disorder*" OR "mental health")) OR (manic* W/1 depress*) OR ((serious OR sever* OR major OR chronic OR longterm OR long-term OR persistent) W/3 depress*) OR ((bipolar OR bi-polar) W/3 (depress* OR disorder*)) OR schizophrenia OR schizophrenic OR psychosis OR psychoses OR psychotic OR "eating disorder*" OR "disordered eating" OR "anorexia nervosa" OR bulimia OR "binge eating" OR "multiple sclerosis" OR "muscular dystroph*" OR "	16,210 results

	peripheral neuropath*" OR stroke OR epilepsy OR epileptic OR migraine* OR "chronic headache*" OR ((learning OR intellectual) W/3 (disabilit* OR handicap*)) OR "mental retardation" OR "mental disabilit*")) AND (TITLE (intervention* OR collaborativ* OR collaboration* OR tailored OR personalized OR personalised OR "demonstration project*" OR "pre test*" OR pretest* OR posttest* OR "post test*" OR (pre W/5 post) OR pre-workshop OR post-workshop OR (before W/3 workshop) OR (after W/3 workshop) OR (before W/10 (after OR during)) OR "quasi-experiment*" OR quasiexperiment* OR "quasi random*" OR quasirandom* OR "quasi control*" OR quasicontrol* OR ((quasi* OR experimental) W/3 (method* OR study OR trial OR design*)) OR ("time series" W/2 interrupt*) OR pilot OR multicentre OR multicenter OR multi-centre OR multi-center OR random* OR controlled) OR ABS (randomized OR randomised OR placebo OR randomly OR trial OR groups OR (intervention* W/6 (clinician* OR collaborat* OR community OR complex OR design* OR doctor* OR educational OR "family doctor*" OR financial OR GP OR "general practice*" OR hospital* OR impact* OR improv* OR individualize* OR individualise* OR individualizing OR individualising OR interdisciplin* OR multicomponent OR multi-component OR multidisciplin* OR multi-disciplin* OR multifacet* OR multi-facet* OR multimodal* OR multi-modal* OR personalize* OR personalise* OR personalizing OR personalising OR pharmacies OR pharmacist* OR pharmacy OR physician* OR practitioner* OR prescrib* OR prescription* OR "primary care" OR professional* OR provider* OR regulatory OR tailor* OR target* OR team* OR "usual care")) OR collaborativ* OR collaboration* OR tailored OR "demonstration project*" OR "pre test*" OR pretest* OR posttest* OR "post test*" OR (pre W/5 post) OR pre-workshop OR post-workshop OR (before W/3 workshop) OR (after W/3 workshop) OR (study W/3 (aim OR aims)) OR "our study" OR (before W/10 (after OR during)) OR "quasi-experiment*" OR quasiexperiment* OR "quasi random*" OR quasirandom* OR "quasi control*" OR quasicontrol* OR ((quasi* OR experimental) W/3 (method* OR study OR trial OR design*)) OR ("time series" W/2 interrupt*) OR ("time points" W/3 (over OR multiple OR three OR four OR five OR six OR seven OR eight OR nine OR ten OR eleven OR twelve OR month* OR hour OR hours OR day OR days OR "more than")) OR random* OR (control W/3 (area OR cohort OR cohorts OR compare OR condition OR group OR groups OR intervention OR interventions OR participant OR participants OR study))))	
3	TITLE (intervention* OR collaborativ* OR collaboration* OR tailored OR personalized OR personalised OR "demonstration project*" OR "pre test*" OR pretest* OR posttest* OR "post test*" OR (pre W/5 post) OR pre-workshop OR post-workshop OR (before W/3 workshop) OR (after W/3 workshop) OR (before W/10 (after OR during)) OR "quasi-experiment*" OR quasiexperiment* OR "quasi random*" OR quasirandom* OR "quasi control*" OR quasicontrol* OR ((quasi* OR experimental) W/3 (method* OR study OR trial OR design*)) OR ("time series" W/2 interrupt*) OR pilot OR multicentre	18,403,364 results

	OR multicenter OR multi-centre OR multi-center OR random* OR controlled) OR ABS (randomized OR randomised OR placebo OR randomly OR trial OR groups OR (intervention* W/6 (clinician* OR collaborat* OR community OR complex OR design* OR doctor* OR educational OR "family doctor*" OR financial OR GP OR "general practice*" OR hospital* OR impact* OR improv* OR individualize* OR individualise* OR individualizing OR individualising OR interdisciplin* OR multicomponent OR multi-component OR multidisciplin* OR multi-disciplin* OR multifacet* OR multi-facet* OR multimodal* OR multi-modal* OR personalize* OR personalise* OR personalizing OR personalising OR pharmacies OR pharmacist* OR pharmacy OR physician* OR practitioner* OR prescrib* OR prescription* OR "primary care" OR professional* OR provider* OR regulatory OR tailor* OR target* OR team* OR "usual care")) OR collaborativ* OR collaboration* OR tailored OR "demonstration project*" OR "pre test*" OR pretest* OR posttest* OR "post test*" OR (pre W/5 post) OR pre-workshop OR post-workshop OR (before W/3 workshop) OR (after W/3 workshop) OR (study W/3 (aim OR aims)) OR "our study" OR (before W/10 (after OR during)) OR "quasi-experiment*" OR quasiexperiment* OR "quasi random*" OR quasirandom* OR "quasi control*" OR quasicontrol* OR ((quasi* OR experimental) W/3 (method* OR study OR trial OR design*)) OR ("time series" W/2 interrupt*) OR ("time points" W/3 (over OR multiple OR three OR four OR five OR six OR seven OR eight OR nine OR ten OR eleven OR twelve OR month* OR hour OR hours OR day OR days OR "more than")) OR random* OR (control W/3 (area OR cohort OR cohorts OR compare OR condition OR group OR groups OR intervention OR interventions OR participant OR participants OR study)))	
2	TITLE (disabled OR disability OR ((physical OR physically) W/1 (disabilit* OR disabled)) OR ((disabled OR disability) W/5 (woman OR women OR female*)) OR "spinal cord injur*" OR paralysis OR paralyzed OR paraplegi* OR tetraplegi* OR quadraplegi* OR hemiplegi* OR "spina bifida" OR "cerebral palsy" OR arthritis OR (back W/1 (pain OR ache)) OR blind OR blindness OR "visually impaired" OR "visual impairment" OR "vision disorder*" OR deaf OR deafness OR "hearing impair*" OR "hearing loss" OR "sensory disabil*" OR "sensory impairment" OR ((serious OR sever* OR major OR chronic OR longterm OR long-term OR persistent) W/3 ("mentally ill" OR "mental illness" OR "mental disorder*" OR "mental health")) OR (manic* W/1 depress*) OR ((serious OR sever* OR major OR chronic OR longterm OR long-term OR persistent) W/3 depress*) OR ((bipolar OR bi-polar) W/3 (depress* OR disorder*)) OR schizophrenia OR schizophrenic OR psychosis OR psychoses OR psychotic OR "eating disorder*" OR "disordered eating" OR "anorexia nervosa" OR bulimia OR "binge eating" OR "multiple sclerosis" OR "muscular dystroph*" OR " peripheral neuropath*" OR stroke OR epilepsy OR epileptic OR migraine* OR "chronic headache*" OR ((learning OR intellectual) W/3 (disabilit* OR handicap*)) OR "mental retardation" OR "mental disabilit*") OR ABS (((disabled OR disability) W/5 (woman OR women OR female*))	2,658,507 results

	OR "spinal cord injur*" OR paralysis OR paralyzed OR paraplegi* OR tetraplegi* OR quadraplegi* OR hemiplegi* OR "spina bifida" OR "cerebral palsy" OR arthritis OR (back W/1 (pain OR ache)) OR blind OR blindness OR "visually impaired" OR "visual impairment" OR "vision disorder*" OR deaf OR deafness OR "hearing impair*" OR "hearing loss" OR "sensory disabil*" OR "sensory impairment" OR ((serious OR sever* OR major OR chronic OR longterm OR long-term OR persistent) W/3 ("mentally ill" OR "mental illness" OR "mental disorder*" OR "mental health")) OR (manic* W/1 depress*) OR ((serious OR sever* OR major OR chronic OR longterm OR long-term OR persistent) W/3 depress*) OR ((bipolar OR bi-polar) W/3 (depress* OR disorder*)) OR schizophrenia OR schizophrenic OR psychosis OR psychoses OR psychotic OR "eating disorder*" OR "disordered eating" OR "anorexia nervosa" OR bulimia OR "binge eating" OR "multiple sclerosis" OR "muscular dystroph*" OR " peripheral neuropath*" OR stroke OR epilepsy OR epileptic OR migraine* OR "chronic headache*" OR ((learning OR intellectual) W/3 (disabilit* OR handicap*)) OR "mental retardation" OR "mental disabilit*")	
1	TITLE (((pregnant OR pregnancy OR (("maternal health" OR prenatal OR pre-natal OR prepartum OR pre-partum OR antenatal OR ante-natal OR perinatal OR postnatal OR post-natal OR postpartum OR post-partum OR pueprium OR puepral) W/3 (care OR service* OR healthcare))))) OR ABS (((pregnant OR pregnancy OR (("maternal health" OR prenatal OR pre-natal OR prepartum OR pre-partum OR antenatal OR ante-natal OR perinatal OR postnatal OR post-natal OR postpartum OR post-partum OR pueprium OR puepral) W/3 (care OR service* OR healthcare)))))	827,127 results

Web of Science Social Sciences Citation Index (Clarivate Analytics)

#7	**#4 AND #3 AND #2 AND #1** and **Review Article** or **Editorial Material** or **Letter** (Exclude – Document Types) and **English** (Languages)	1,981
#6	**#4 AND #3 AND #2 AND #1** and **Review Article** or **Editorial Material** or **Letter** (Exclude – Document Types)	2,002
#5	#4 AND #3 AND #2 AND #1	2,281
#4	PY=(2012-2025)	4,820,066
#3	TI=(intervention* or collaborativ* or collaboration* or tailored or personalized or personalised or "demonstration project*" or"pre test*" or pretest* or posttest* or "post test*" or (pre NEAR/5 post) or pre-workshop or post-workshop or (before NEAR/3 workshop) or (after NEAR/3 workshop) or (before NEAR/10 (after or during)) or "quasi-experiment*" or quasiexperiment* or "quasi random*" or quasirandom* or "quasi control*" or quasicontrol* or ((quasi* or	2,091,996

	experimental) NEAR/3 (method* or study or trial or design*)) or ("time series" NEAR/2 interrupt*) or pilot or multicentre or multicenter or multi-centre or multi-center or random* or controlled) OR AB=(randomized or randomised or placebo or randomly<br>or<br>trial or groups or (intervention* NEAR/6 (clinician* or collaborat* or community or complex or design* or doctor* or educational or "family doctor*" or financial or GP or "general practice*" or hospital* or impact* or improv* or individualize* or individualise* or individualizing or individualising or interdisciplin* or multicomponent or multi-component or multidisciplin* or multi-disciplin* or multifacet* or multi-facet* or multimodal* or multi-modal* or personalize* or personalise* or personalizing or personalising or pharmacies or pharmacist* or pharmacy or physician* or practitioner* or prescrib* or prescription* or "primary care" or professional* or provider* or regulatory or tailor* or target* or team* or "usual care")) or collaborativ* or collaboration* or tailored or "demonstration project*" or "pre test*" or pretest* or posttest* or "post test*" or (pre NEAR/5 post) or pre-workshop or post-workshop or (before NEAR/3 workshop) or (after NEAR/3 workshop) or (study NEAR/3 (aim or aims)) or "our study" or (before NEAR/10 (after or during)) or "quasi-experiment*" or quasiexperiment* or "quasi random*" or quasirandom* or "quasi control*" or quasicontrol* or ((quasi* or experimental) NEAR/3 (method* or study or trial or design*)) or ("time series" NEAR/2 interrupt*) or ("time points" NEAR/3 (over or multiple or three or four or five or six or seven or eight or nine or ten or eleven or twelve or month* or hour or hours or day or days or "more than")) or random* or (control NEAR/3 (area or cohort or cohorts or compare or condition or group or groups or intervention or interventions or participant or participants or study)))	
#2	(TI=(disabled or disability or ((physical or physically) NEAR/1 (disabilit* or disabled)) or ((disabled or disability) NEAR/5 (woman or women or female*)) or “spinal cord injur*” or paralysis or paralyzed or paraplegi* or tetraplegi* or quadraplegi* or<br>hemiplegi* or “spina bifida” or “cerebral palsy” or arthritis or (back NEAR/1 (pain or ache)) or blind or blindness or “visually impaired” or “visual impairment” or “vision disorder*” or deaf or deafness or “hearing impair*” or “hearing loss” or “sensory disabil*” or ”sensory impairment” or ((serious or sever* or major or chronic or longterm or long-term or persistent) NEAR/3 (“mentally ill” or “mental illness” or “mental disorder*” or “mental health”)) or (manic* NEAR/1 depress*) or ((serious or sever* or major or chronic or longterm or long-term or persistent) NEAR/3 depress*) or ((bipolar or bi-polar) NEAR/3 (depress* or disorder*)) or schizophrenia or schizophrenic or psychosis or psychoses or psychotic or “eating disorder*” or “disordered eating” or	524,697

	"anorexia nervosa" or bulimia or "binge eating" or "multiple sclerosis" or "muscular dystroph*" or "peripheral neuropath*" or stroke or epilepsy or epileptic or migraine* or "chronic headache*" or ((learning or intellectual) NEAR/3 (disabilit* or handicap*)) or "mental retardation" or "mental disabilit*") OR AB=(((disabled or disability) NEAR/5 (woman or women or female*)) or "spinal cord injur*" or paralysis or paralyzed or paraplegi* or tetraplegi* or quadraplegi* or hemiplegi* or "spina bifida" or "cerebral palsy" or arthritis or (back NEAR/1 (pain or ache)) or blind or blindness or "visually impaired" or "visual impairment" or "vision disorder*" or deaf or deafness or "hearing impair*" or "hearing loss" or "sensory disabil*" or "sensory impairment" or ((serious or sever* or major or chronic or longterm or long-term or persistent) NEAR/3 ("mentally ill" or "mental illness" or "mental disorder*" or "mental health")) or (manic* NEAR/1 depress*) or ((serious or sever* or major or chronic or longterm or long-term or persistent) NEAR/3 depress*) or ((bipolar or bi-polar) NEAR/3 (depress* or disorder*)) or schizophrenia or schizophrenic or psychosis or psychoses or psychotic or "eating disorder*" or "disordered eating" or "anorexia nervosa" or bulimia or "binge eating" or "multiple sclerosis" or "muscular dystroph*" or "peripheral neuropath*" or stroke or epilepsy or epileptic or migraine* or "chronic headache*" or ((learning or intellectual) NEAR/3 (disabilit* or handicap*)) or "mental retardation" or "mental disabilit*"))	
#1	(TI=(((pregnant OR pregnancy OR (("maternal health" OR prenatal OR pre-natal OR prepartum OR pre-partum OR antenatal OR ante-natal OR perinatal OR postnatal OR post-natal OR postpartum OR post-partum OR pueprium OR puepral) NEAR/3 (care OR service* OR healthcare))))) OR<br>AB=(((pregnant OR pregnancy OR (("maternal health" OR prenatal OR pre-natal OR prepartum OR pre-partum OR antenatal OR ante-natal OR perinatal OR postnatal OR post-natal OR postpartum OR post-partum OR pueprium OR puepral) NEAR/3 (care OR service* OR healthcare))))))	87,839
